# Supplementary material for: Population genetics of the freshwater fish Prochilodus magdalenae (Characiformes: Prochilodontidae), using species-specific microsatellite loci
Source: PeerJ. 2020 Nov 11;8:e10327. doi: 10.7717/peerj.10327 (PMC7666565; doi:10.7717/peerj.10327)
Supplement: Supplemental Information 1 — 1: main channel of the river; 2: Floodplain Lake [file peerj-08-10327-s001.docx]

| Hydrographic Area | River | Section | Sampling Site | Code Map | Municipality | Department | Coordinates | |
| --- | --- | --- | --- | --- | --- | --- | --- | --- |
| Magdalena- Cauca | Cauca | S1 | Bolombolo^1^ | 1 | Venecia | Antioquia | 05°58´05.00” N | 75°50´24.97” W |
|  |  |  | Puente Real^1^ | 2 | Santa Fe de Antioquia | Antioquia | 06°30´51.96” N | 75°49´04.49” W |
|  |  | S2 | Gurimán^1^ | 3 | Briceño | Antioquia | 07°09´22.32” N | 75°37´08.00” W |
|  |  | S3 | Espíritu Santo river^1^ | 4 | Yarumal | Antioquia | 07°15´03.45” N | 75°26´16.75” W |
|  |  |  | Puerto Valdivia^1^ | 5 | Valdivia | Antioquia | 07°17´11.90” N | 75°23´43.59” W |
|  |  | S4 | Cáceres^1^ | 6 | Cáceres | Antioquia | 07°34´56.93” N | 75°20´56.21” W |
|  |  |  | Man river^1^ | 7 | Caucasia | Antioquia | 07°57´22.93” N | 75°12´28.06” W |
|  |  | S5 | Margento^1^ | 8 | Caucasia | Antioquia | 08°02´04.21” N | 74°57´14.74” W |
|  |  | S6a | Grande^2^ | 9 | Montecristo | Bolívar | 08°17´46.45” N | 74°30´04.93” W |
|  |  | S6b | Caimanera^2^ | 10 | Montecristo | Bolívar | 08°17´21.04” N | 74°30´05.24” W |
|  |  | S6c | Guaranda^1^ | 11 | Guaranda | Sucre | 08°27´55.81” N | 74°32´05.08” W |
|  |  |  | Achí^1^ | 12 | Achí | Bolívar | 08°38´29.55” N | 74°30´55.15” W |
|  |  | S8 | Panela^2^ | 13 | Pinillos | Bolívar | 08°52´51.75” N | 74°27´09.74” W |
|  | Magdalena | Lower | Palomino^1^ | 14 | Pinillos | Bolívar | 08°55´09.20” N | 74°26´21.50” W |
|  |  |  | Pijiño^2^ | 15 | Santana | Magdalena | 09°19´45.60” N | 74°27´15.60” W |
|  |  |  | Mompox^1^ | 16 | Mompox | Bolívar | 09°14´12.00” N | 74°27´11.30” W |
|  |  | Middle | Llanito^2^ | 17 | Barrancabermeja | Santander | 07°10´14.30” N | 73°51´03.50” W |
|  |  |  | Barrancabermeja^1^ | 18 | Barrancabermeja | Santander | 07°03´09.67” N | 73°52´41.65” W |
|  |  |  | Chucurí^2^ | 19 | Puerto parra | Santander | 06°49´38.94” N | 74°03´36.14” W |
|  |  |  | Río Viejo^2^ | 20 | Cimitarra | Santander | 06°33´48.29” N | 74°17´28.04” W |
|  |  |  | Puerto Berrío^1^ | 21 | Puerto Berrío | Antioquia | 06°28´18.76” N | 74°24´26.47” W |
|  |  |  | Palagua^2^ | 22 | Puerto Boyacá | Boyacá | 06°04´02.79” N | 74°30´56.26” W |
|  | San Jorge |  | San Marcos River^1^ | 23 | Río San Marcos | Sucre | 08°39´46.50” N | 75°08´07.00” W |
|  | Cesar |  | Mata de Palma^2^ | 24 | El Paso | Cesar | 09°33´33.30” N | 73°38´44.10” W |
|  | Nare |  | Samaná Norte river^1^ | 25 |  | Antioquia | 06°14´31.33” N | 74°42´53.62” W |
| Caribbean | Sinú |  | Caño Grande^1^ | 26 | San Bernardo del Viento | Córdoba | 09°23´00.76” N | 75°53´09.98” W |
|  |  |  | Doctrina^1^ | 27 | Lorica | Córdoba | 09°17´01.28” N | 75°53´40.34” W |
|  |  |  | Beté^1^ | 28 | Medio Atrato | Choco | 05°59´42.19” N | 76°46´21.23” W |
|  | Atrato |  | Palo Blanco^1^ | 29 | Vigía del Fuerte | Antioquia | 06°10´05.59” N | 76°42´05.54” W |
|  | Dams |  | Urra I | D1 |  | Córdoba | 07°56´24.00” N | 76°17´23.99” W |
|  |  |  | Riogrande | D2 |  | Antioquia | 06°40´47.79” N | 75°35´30.30” W |
|  |  |  | San Lorenzo | D3 |  | Antioquia | 06°22´55.68” N | 74°59´57.61” W |
|  |  |  | Playas | D4 |  | Antioquia | 06°19´03.00” N | 74°57´38.00” W |
|  |  |  | El Peñol | D5 |  | Antioquia | 06°14´25.74” N | 75°12´39.35” W |
|  |  |  | La Fe | D6 |  | Antioquia | 06°06´02.60” N | 75°29´39.06” W |
|  |  |  | Miel | D7 |  | Caldas | 05°33´36.74” N | 74°53´15.87” W |
|  |  |  | Muña | D8 |  | Cundinamarca | 04°30´49.19” N | 74°15´00.76” W |
|  |  |  | Calima | D9 |  | Valle del Cauca | 03°53´40.47” N | 76°29´41.52” W |
|  |  |  | Río Prado | D10 |  | Tolima | 03°47´10.59” N | 74°50´54.91” W |
|  |  |  | Betania | D11 |  | Huila | 02°41´25.89” N | 75°28´35.51” W |
