# Supplementary material for: Population genetics of the freshwater fish Prochilodus magdalenae (Characiformes: Prochilodontidae), using species-specific microsatellite loci
Source: PeerJ. 2020 Nov 11;8:e10327. doi: 10.7717/peerj.10327 (PMC7666565; doi:10.7717/peerj.10327)
Supplement: Supplemental Information 4 — Forward and reverse primers are indicated in gray. Microsatellite motifs are highlighted in green. [file peerj-08-10327-s004.docx]

**Locus Pma01**, motif ATCT (reverse complement: AGAT)

>IFVYH3A01CP0FT length=629 xy=0999_1063 region=1 run=R_2013_09_11_14_57_29_

GCAGGGTGCTGTGAGGTAAATTAGTCCCAAAGGAGGACTTGTCATTTCCCGGTTTTCCCATCATCAACATTCCATATAAGTGTTACTGGTGGGATAGATAGGACATTCATTTATGACACCACTATCTGGCCAGTGGGCCAGTGAGCCATCACCCCATGTGGGGTTTAATGCCCGGTCTGGGCTGCACTAGATAGATAGATAGATAGATAGATAGATAGATAGATAGATAGATAGATAGATAGATAGATAGATAGATAGATAGATAGATAGATAGATAGATAGATAGATAGACCTGCATGGTTATCCAAATTACAGCTGGGCCAATGTAGTGTTTGCTGTGGTGGTTGTTGTGTCTCAGTGGTTAACACCGCTACCCACCAGCGAGCTACCACCCCTAGGTGGCAAATGCTGAGCTACACCAACAAGAGACCTTGAGCAAGACTCCTAACACTGGCCTAAAATGTGTATATGTGTGTTGCAGACATGGTGAACCCTATTTCCCATCACCACCACAATAAACAGCAATTAAAATAATTAATCGAATAATTAATGGAGTAACAGAGTTAAATTTTGCCCTCAATATATACATTTGCGGTCTTACTATATTATCTGGTTGCCTCAATATATTA

**Locus Pma02**, motif ATCT (reverse complement: AGAT)

>IFVYH3A01EX11E length=550 xy=1911_0064 region=1 run=R_2013_09_11_14_57_29_

CAAACGACATTCAACATGACAGTGCTGTGGTGCTGTAGTTCTTTGACTTTGTCAACATTTCCAGATAGATAGCTAGATAGATAGATAGATAGATAGATAGATAGATAGATAGATAGATAGATAGATAGATAGATAGATAGATAGATAGATAGATAGATAGATAGATAGATCACAACATCACAGCAGTTACCACTGTGCCTAACATTACAAAATTATAAATGTAGTTGTCTTTGAATATTAGCGTGTGCTCACTGCAGTTTGCATCAATTTGGTGGGATGTACCATTTAAGCCCACCTGTAAAAAAAGCTAATTATTTAAATTAATCAGCTCTACACAACAATTTATGATGCATTTATCTAAATATCAATGTTAAATGAATAACTAAATGCTTACTATTTGTAGCAGAAAATACTAATTCTTGTGGCAGTGTAGCATTAACAAGGCTAGGGGTGTTAGCATGAGATTAGCTATGTTTGCTAGCTCATAAAACTCACCCTTTCATTTGAAGGGAAATAGCATAATTAGGCAAAGTACTATTTATTTATATAC

**Locus Pma03**, motif ATCT (reverse complement: AGAT)

>IFVYH3A01EASA0 length=675 xy=1645_3898 region=1 run=R_2013_09_11_14_57_29_

ACAGTAAATGATACCCAGCTATTTCAGTCCACAATGATCTCTAACACTGATATCCACATTTAGAGCCAGAGTGATGGGTCATCTATGAAATGGTTCTATATAACACCAAAGCCATTGCAGGTCTTGTTCTGCCCTTTATTCCAACTCACCTGAGGCCCCAAACAGTGTGGAACATAGATAGATAGATAGATAGATAGATAGATAGATAGATAGATAGATAGATAGATAGATAGATAGATAGATAGATAGATAGAAAGTATTGATGTGTGAAATTTGCATATTGTTACACCCCATTGAGTGGGTTCAGGTACTTATGTAAATGATGTCCAGTGCTCCAAGGGACACTTGGCACTAGGCATGGTGACCCTATAGGCTCATGTGTAGCAGTTTCAGAAGATCCTATTCGATTGGTATTCCCAAAAACAGTTGTTCCTTGATGGAAGACCTAATAAGTTACACAGTGATGTTTATAAATAACGTTTATGTCTGTTGCAAGTGTCTAAGTCATGTGAAAATAGCTTTGACTACATAGCACAAAAGCACAGTCAGTCATAGTGCCAAAGAGGAGTCTGAGGTTAGTGTACTGCTGAGAGAGAGGAAATGGCCTGCCTCAGGTGTGTACAGAGCTGGAAAAATAAGGGCTGTCTTGGGTCCTGTTGGGCTGGAATGTAGATC

**Locus Pma04**, motif TGCG (reverse complement: CGCA)

>IFVYH3A01AUQYW length=420 xy=0233_0374 region=1 run=R_2013_09_11_14_57_29_

CTGCTGTTCTACAGAATAAGGATTTACCATCATCTCACATAACTGCATGCATGCAATGATGAGGCAATAATGTAAAAACAGTGGTCATGAGGCCTCAGGTCTTCTTCCAGACAGTCTGTGTATGTACCTATGATGCTGGTTAACAACAAGAAAACACTGACAGGTTGGCTAATTATAGCAGCCCATATCAAATAGCAGAGCCATGCAAAACTGAGTAATCTGACAGCCTGTATTGTGTTGCCTTGTCTGTCTGCTTGGTCTTGGACTGCTAAAGGTCATAACCGCACACACGCACGCACGCACGCACGCACGCACGCACGCACGCACGCACGCACGCACGCACGCACGCACGCACGCACGCACGCACGCACAGATGAAACTCCAGTGGTCGAACCTGTGCCCAAGCAACTGTGACGTGCT

**Locus Pma05**, motif AATC (reverse complement: GATT)

IFVYH3A01B1NJU length=415 xy=0721_3304 region=1 run=R_2013_09_11_14_57_29_

ACAATGCACAGGGTACTATTTGAGCACAGCCCGTAATCCAATAGAAGTACTGTATCATCGTGACCTGAATTGTGAAGCCCACCAACATCCCTGAGACCCCATACCTGCTTATATGGATTTTGACTTTGTCCACACTATCAACATTGTTAAAGTAATGACTCAACACACTAACATGAGCATGCCAGCCCAGAAGTACTTAGACTCTGTGTATACATGGTGTTTTCTTTTCCTTTATGTAAACTGCTTTTCTGTAAAGCGCTATAGAAATAAATATTGATTGATTGATTGATTGATTGATTGATTGATTGATTGATTGATTGATTGATTGATTGATTGATTGATTGGGGTGATAAATCTCGTTAAATGGAAATGTCCAGAGAAGAGTATTACGAGCATGCTTGTTATGCAGAAGTAT

**Locus Pma06,** motif ATCC

>IFVYH3A01DTPGO length=466 xy=1451_1686 region=1 run=R_2013_09_11_14_57_29_

GCTGACGAGCTCAGTATCCTGTCTGAACTCCCTCGCGAACAGAGCTGGCCTTGATCCGAACTCCGCCCTGCGGAATTCCGGCCAGGATCAGATTCCAGCTTTGCATGTTGCGCGGCGTTCTGGTCTAATCTCGGGTGACATGTCATCCTCTACCGACCTCCAGCACGAGCCCACAGAGTGGCCTTTGTCCCGCAGCTCAGCCAGCCGTATCTCCAGCCGTCCAGCCTCAGACGCTGCTCGCTCCGCCCAGAGCCTGCTGATGGATAGCCAGAGTCCCACCCGGTCCACGGCTTCCATATCCTCCGCAGAGCCGCGCCGGGCGTCCATCCATCCATCCATCCATCCATCCATCCATCCATCCATCCATCCATCCATCCATCCAACCACTCCTCTGCAGAACACACCGTCATTTTCTCCAATTTATTGAGAAACAATATCGAACAAGTGCTAAAACCCGATTCAATCC

**Locus Pma07**, motif ATCT

>IFVYH3A01E4HEH length=486 xy=1984_0903 region=1 run=R_2013_09_11_14_57_29_

ACCTGTCAATGGAGGAGTTAAGATGCATATCATCTTCCTCACAGCCTTATATACACTCTTAACTCTTTTCATTTCCCCCCTCTGTCTCTCCTGTCCCCTCTCACTCTCTCTCTACTTCTCTCCATCATTCCCCCTACTATCTCTGTTTCCTTCTACCTTCTCTTTATCCCTTTCTCTGTATCACTCTCCCCATCTATCTATCTATCTATCTATCTATCTATCTATCTATCTATCTATCTATCTATCTATCCTCACTCGCCCATCTATCCCTTTAACCCCCCCTTTCATTCTCTCTCTGTCTCTTTCTCCCTATGTTCCGCCTTTATCCCCCTCTGTCTCTTTCTTTTATGCCCTCCCCCTCCTCTCTCTCTCTCTCTGCACTTCTTGGCAAATCACAGGGCATCAGCTGATATCTGTAGCGGAAAGGATTTGGTGTTCCACGAAGTCTATTTAAAAGTGCCAATATAGCTCTCATCTCTCTCTCTC

**Locus Pma08**, motif AAAG (reverse complement: CTTT)

>IFVYH3A01DJH33 length=411 xy=1335_0737 region=1 run=R_2013_09_11_14_57_29_

GTTTTTATTATTCCCCATTTTCTCCCAAATTTCTCTTTCTTTCTTTCTTTCTTTCTTTCTTTCTTTCTTTCTTTCTTTCTTTCTTTCTCAGGGTTCTCTTCTGTTTTGATGCTGCAGTCTGTCTCTCAGGGATCTCTCAGCAGTCTGCTGCTGTCCGGAGTCCACAGTTACAGTCACAGTTCCACACACAATCGCACACACTCACCTCCACATGTTCATACACACACGCCCTCCCCTCCGCCAGGACTCGCACCTCTGCACAAACCAGTAAAACGCAGAACAGCTCAAAACCCATGTACGTATCTGTCTAGAATTACTACTAACCTATCCATATACCTCCAACAAACACATTCTGTATCACCTTGGCTTGGATATTTTTTCTAGTGTCCCGTCCAGCCTGGCAAAAATGTT

**Locus Pma09**, motif AAAC

>IFVYH3A01AZAA5 length=359 xy=0284_3167 region=1 run=R_2013_09_11_14_57_29_

TATAATACAAAAATATAAATACTGCAAACACAAATGTAAAAAATAATAGAATATTAAAAAATGAATAACAATAGTTGCCAGCCATGTTTCTCTCCTGTTTCTGCCAATGTGACATGTAGGAGAACACAAACAAACAAACAAACAAACAAACAAACAAACAAACAAACAAACAAACAGCTCACACAAGCAGTGGACAGAACGCCGGGAACTTCAGGAACAAAGAGACAGAACGCCGCATGTCCGACAGGAAGCCAGACACACTTGGAGGGGGTGGGAGTGGAGGGTGGGGGGCGCTGGCAAAAAGGTAGACGGCATACACACTCAAGACACGTGTACACACACACACACACATACACACA

**Locus Pma10**, motif ATAC (reverse complement: GTAT)

>IFVYH3A01EG7DO length=834 xy=1719_0266 region=1 run=R_2013_09_11_14_57_29_

ATTGTACAAGCATATGCCATGCATTCTAAATGTCATATACAGTACAAGTGCCAAGAATTACAAGAGTTATAAGTGTTATGTATGTATGTATGTATGTATGTATGTATGTATGTATGTATGTATATAAGTTATAAGTTATAAATGCATTTGTACTGCATTATTAATCCTAAGGATTCATGAGAATTCATGTACAGCGTGGTCCTATTTTATATGTGCTAAATATTTAATTTTCTATATACAGTTTACATGAGCAAAGTAGCTTTATAGGGGGCGCTACATAACAGCAGGCATTAGTCAAAACACAGGCAGGCAAGTACATACAAGGCAAAACCATACTCAAAAATATTACAGGTTTTGAATGCTGGCAGGCTCTGTCTCTGACCACCTGTAAAATGTGGTCTAAGTGATCCGATCATAATCCAATCACAGTAAGCCCTGGTGTTGACAACTGGCTTTCCATGTAGTATAGTGAAATATGAACATGATCTGCTCAGGGAAACCTGTGTTGATGCACTGTATAAACAGGCTCAAATGTGTTTGAAAAGTCCACAGATAGATTAAAGACCACATGATGAACAGCAACAAGCCATTAATAAAGTGCTGAGCTCAGTGAGCCTCAGCACTGCGCAAACTCAACAGATTTTTTTTAATGATTATTATGATCATGTCTGTGACCTGAATCCTCATTTATTTTTGGTGTTTTAATTAACAAACAAGTTTAACCAAGCTTGCAAATTCTGACACCACCCGCCTTGGCTTTGTCTGAGGGCTGCTGCAGGTGCTGGATACAGATTTCATGACCTCCTTGCTCTCAGGCATGCGGCCCAGTTCCTA

**Locus Pma11**, motif ATCC

>IFVYH3A01E111H length=610 xy=1956_2371 region=1 run=R_2013_09_11_14_57_29_

AGAGTGCTCTGAACAAACTGACTCTGTTGTAGGAGTTGAAGCTGAACTCTCTGGAGATTGTGGTGGAACAAAGGACTCTCAACAAGCTGTTAGCCATCTTAGACAATACCTCACACCGTGTGGATACTACACTGGAACAACAGCGAAGCAAATTTAGTAGTGGACTGTTACAGCTGCGCTGTGTTAACAAGCGCCATGTGAGATCTTTTTTTTTCATCCATCCATCCATCCATCCATCCATCCATCCATCCATCCACCACCATAGAGCAGGTATGATTTGGGTGGTGGATCATTCTCAGGGATGCAATAACCCTAACGTGGCGGTGGCATGTTAGTGTGTGTTGTGCTGGCCCAGCTGGACTGGTAATTTGGTGTTCTGGATATCTTACAGGTGGGCCGATGCACCTTTAGGCTGATATAATTTATTTTCATAATTTAAAAAAACTGTATAGTTTTGAAAATGTACAGGGCCAGATCCATTAAATAGGGACAGTGACCCCTTTGGTCATTTTCTATGCTGACGCTGGACGGATCCAATCATACCTCTCACTGGACCCTTCCCCTCCCCCCCGACTGTCTTCCCTGTTTCTAGTATCAGATGACTTCCCCC

**Locus Pma12**, motif ATCT (reverse complement: AGAT)

>IFVYH3A01D6OJM length=554 xy=1599_0816 region=1 run=R_2013_09_11_14_57_29_

ATGGAATTCCCCTTCCTCTTGCTAAATGGAGCAGTGGAGTGCACATTAGTAAAAGCCACCTCAAGCGTTGTTATTGCTAATACAGTGTACTTGAACAGAAACTTAAGTTTTGACTCCATTTTACTGTAGAGGCTAACATGTCCATCTGTTTAATCATCCCTGCTTCTAAAGAGCTTACGTGATTTGTCTAAATTGTGTATTTACATGATGGTTTGCTCATTAGCAGACTGTTTGGCAAAAAGAAATGTTAGGAAATGCTTCTTCTCAGAGCCAGATTGCTTTGGCAAACCTTGCAGGAGCTCTAATGTGCTGTTTATGTGAGGTCTATAGAGTTTTAGAGTATTTAATGCATGGGCTTCTGTTGCTCATGTAGGCTACTTGCTTTTTGCAAACAACAATTAGTTCTGACTGCATAGATAGATAGATAGATAGATAGATAGATAGATAGATAGATACTTTATCAGATGAAGTAATTTAGATAAAGATAGAATAAAAATAGNTAAAAATGTCCTACCTGTGTGTAGGGCTACAGAATGAGACAATGGAATGACATC

**Locus Pma13**, motif AAAT (reverse complement: ATTT)

>IFVYH3A01D3U1J length=670 xy=1567_0341 region=1 run=R_2013_09_11_14_57_29_

TCCCAGCTGTTACTGGTGAGGGGTTACACGGTGTTAGGCAAGGTTCAGTCACAGGGGACACATTGTAATGGACAAATGACCACAACAGTTAATATTAGGGTTTGTCCCCTGAAAGACATTCACCTGTGATTGGTCGCTAATGGCATCAGGAGCACCTGGGTTACCTACTGCAGCCTTAAGGCTCTAAGACGTGTATCCAAACTTTTGACTGCTAAAACACAATGCGCCTTTACAGCAGCTCATAGATCTCTAGGCTGTGGATCGCTATAGTCCGTACATAGGTGATCTGCTAGGCTGCAGTATTCCTTCCCAGCCCCTTCGTTTACGCTGAAACGCCGGAATACTGAGAGAGAGACGAAGGACGCCATGACATTCGGCTCTTTTGGGAAATATCTGTTTTGTTTCGACCCCTCTTTAATCAAATCTACCGGCAGTGGTGACCCAAGGGGGCCGAAGCTATTTACCCAGCGAAAAACATCTTTATTCATTATTTATTTATTTATTTATTTATTTATTTATTTATTTATGAGCAGGCTGATGCCCATCTCTGCCTAATTAGAATCACTTCACCACAGCCTTTGAGTAATAACGTGGCATGCATATTCCCAGAACAAGCGGGAGCACGAGCATATTTCATAGGCTTGGCTCTATTATGGACACGGCGTGCGGA

**Locus Pma14**, motif TTC

>IFVYH3A01CFJM1 length=585 xy=0880_0155 region=1 run=R_2013_09_11_14_57_29_

TCATATTCGATAGGAGTTCCTGGCTCCTCATCCCAATTAGAGCGGACCCCGAAACATCGGCACAGCGGGGTCTCTCGCTGGCCGCTATCAGGGCAGGATGGATTAGGGGAAGTGTGAAATGACCTTGTGATTCTGAAGTTTAAAGACGTGGTTAGCCCCTGGCTCAGAGCGACGGCGGGCATGAGGGGCAGTGTTCTGCATTCCGATTTCGTCCTGCAAAGGTAGCCCGCCCTTCCGAATCCGCTTACGGGAATGAGCCAGACGGGCAGTACGTGGGCCTGTTCAGGGTCCTGCTGTTCCTAGCTTCTCATGCTAACTTAATTAGCAAGTACGCAACACTGTTGTTATCCTTCATCTTCTTCTTCTTCTTCTTCTTCTTCTTCTTCTTCTTCTTCTTCTTCTTCTTCTTCTTCTTCTTCTTGTGTGATTGTTTTGTTCAGTTTTTGAGATATTGGCAATGTTCCAACACCGAAATGTTCGATCTGATTGGTTATGGTGTGCTTGTATACGGCACCCACTAAGAATTAGCACGGTCGGTACCAGTACCAGCCCTATCTACTGGTACCTCTGCACTACGGACATCTC

**Locus Pma15**, motif ATT

>IFVYH3A01DX7I3 length=760 xy=1502_2829 region=1 run=R_2013_09_11_14_57_29_

AATGAATATACAGTTCCTGGTTCTGTGCAGAATTTCTTGTAGAGTACCACAGGGGTCAGTTTTAGCTAAACTGATGACACACGATTGTACAGTTCCTAGACTTGGTAGACAGACTTAGAAGCTGTTTGTTAATCATTTTAATCATTTGAAATATTTGGCTAAGGTTCAGCCTTTTCATTCTCAGAGCGAGACAGAATAACCTGGTCATGAGTTTGTTCATGAGTCAGCAGAGTTGATCACTGTAATGGTCCTCTTACTGGGGCTCCTAAGACAGCAACCCATTCCCTGCAGCTTCTACAGACACTGCTGTTATTACTAATGACTGTCTGTGAAGCACTTTCAGAAGCCAACATTATGAAATCTGCCCCATGAATAAAGATTATTCAGATTGTTTTATTATTATTATTATTATTATTATTATTATTATTATTATTATTATTATTATTATTATGTGTTATTATAGGCTTCATGTCATATCATCTAGTATCCAAATATCACAGCAGAATGATACGACTGCTCTGTAGCAGCTCCATGGTTAATCCAGGAAACACATCTGGAGGTTCCTCCTTAATCATGTTAAATGAGAAGTATGTAAATGTCTGGTCCAGCAGCAGCGCTGAAAGTCAGCCACTGTCACAATGTGTATATAACACAGATCAACACTCAGACCTACTCTAACCAGCCCACATGCACACAGAAGCTTTCAGAGCAACCATGAGCTGGACCTCTCTGCTGCTGGTGGCCTTCTTACCATGTAGGT

**Locus Pma16**, motif ATT

>IFVYH3A01CZBTJ length=478 xy=1105_1541 region=1 run=R_2013_09_11_14_57_29_

CAACTGTCTGTAAAGCAATTTCAGCTGTCAGTATGAAACGGTTTGAAAGGGTCTCTATAAATAAACATTATTATTATTATTATTATTATTATTATTATTATTATTATTATTATTATTATTATTACTTTGTCTATTGCTGCCTATATATGTGCTGTCTTGTAGGTACTGTTGTGACATTGGCCTGGCCCAACATTTGGCCCTGACAACCTGATGAGACAGCATAGCGACCAGCCCTGATTTCCTTCACACTATGGTCTTTGGCCACGCCCACATGCAGTCACTCCTTTTAGCCAATCACCGACGTGTTTACAATGATGTGTTACAAAGACATTTGTCCAGTGTTATAAAGGCTTTATAGAGCTTTATGAATGCACTTATAAGGCATTATGAATTACCCAATTAATAAAAAATGTGTTATCAACACAAATGGCTAATATCTAACAGCATATCAGCCAAAAAGCCCCAGCATGTCCTTATT

**Locus Pma17**, motif ATT (reverse complement: AAT)

>IFVYH3A01B7ZME length=344 xy=0793_3972 region=1 run=R_2013_09_11_14_57_29_

AAAATGTGTGATGTTTTATGTGTATTTCTGTGTATTTCTTTCTGTGGGCAGCAAAGTGCTTGAGCTGTTTGAGCATAACAAACTGCTTTTAGATGATCTTAAGTGAAGATTATAAAAGCTCATTTTAAAATATCAAAATATATCTCAGTATGTCAGATCTGAATGGCAATTGTGGAAGTTTCAGTAATAATAATAATAATAATAATAATAATAATAATAATAATAATAATAATAATGTTTATTTATAGAGCACCTTTCATACCGTTTGAAGTGGCTCAAAGTGCTGTACAGACACACACACACTTAAATACCATGTTAATGTAGTTTTTAGTAAATGCTGAGTC

**Locus Pma18**, motif ATT

>IFVYH3A01EZ8RM length=608 xy=1935_3792 region=1 run=R_2013_09_11_14_57_29_

CATTATTAATACAGTGTAAAACAGGACTGAGACAAAACCCGGAGGACTTTTCATTGACACACACGGAGAGATCTGAACAGAAAAAGAGACGAGAAAAGAGTCTAATGTCAGAAAAGTTTAATGTCTATTTAACATGTGACAGAAATACATAACCATCAGACAATTAAACTTAGAGTAAACATTATTATTATTATTATTATTATTATTATTATTATTATTATTATTGATGTTAATATCCTGATGGTGGGTGTATGAAGTGATTATGGGTCTGTTAAATACAATGAGATTATTGAGGACTCATTGATGTTCTGGTCTCTGCCAGTTTCTAAGAAGTGGTGGTCTACATGTCTTAAGTGGGGTTTACTAAACCTGTGGTCACTCCAGGCCCAGCATTTCTGTTCACCTCTTTAATCCAGACTGGTAGAAATGTTTACTGATCAACTCCTGATAGTGATCATCTCTCCACCTTCCTTCACCATTATTGCTGAGTCAGGCTGTTTCGCCGCCTCTTTCTCTGTTTAGATCTCTTTGGTGATATTGGCATGTGTGGATGAATGAAATGAGATTAAAGGATCTGTGTGTATCGTGTCCGTCCTGTTCTAGATTTC

**Locus Pma19**, motif AAC

>IFVYH3A01CKI9E length=547 xy=0936_3568 region=1 run=R_2013_09_11_14_57_29_

ATAACGTGACCGGCTTACAAGAAATCTCTACACAGTATAACACAACACATGCTAATGTAATACAAGCAAACATGACTGATTCTACACTGTTAAGTACAGATTCTAACAAAACTAACAATCCAAACTTTAATACAACAACAACAACAACAACAACAACAACAACAACAACAACAACCACCCCTTCATACACAGCATCTACAACACAGTTCAGCTCAACAATCTCAGAAAATACAACACATTATGGCATGCCAAATAATCTAAACACAACACTGACAAATACAACTGCAGTGCCTGTGAACACTGCACAGCACAATACAGCAGTACCTCTGAATGCAACTACCCCCCGGACTTCTGATACAACACAGCCTAATACAACTACAGATGCAACTGCATTACCAAGCACCACACGTGCCTCAGTTTACACAATGGATCATCCAAATGTAACAGCATCGGTGAATACAATACTTCCAAACAGCACCGGGTCACCACCTTACACAAATGTATCAGCACCTGTCAGTATAACACATGCAGACACCACTACCACAAA

**Locus Pma20**, motif GTT

>IFVYH3A01B5BFK length=453 xy=0763_2190 region=1 run=R_2013_09_11_14_57_29_

ACACTACCCCCTTTCCACTGCTATGGGGGGTTGACCGGTCAGTTTTTTTTTTTTTTTTTCATTTGTTTATTTTTTTGTTGAAAATCAATCTGTTTACAATCTGAATAAATTATACTTTGGATTTGATTTAGTACACTGAACAAGGCCTTAAAGACTCTCCTAAGCTAAAATTCGGATGTGAATACCACATATAGAGTAGTCATTATCTAGAGCAACAAAGTAGAAAGTTATTTAAAAGATGTAAAAAGTTCACAGAGAATAAAAACCCATTTAGACTAAGGCCGTGGCTTGCTTGAATGCAATGCTATCTGCTAAGCTATGGCCTAACCACTGTCTGGGCCCCTGATGATGTTGTTGTTGTTGTTGTTGTTGTTGTTGTTGTTGTTGTTGTTGTTGTTTTTTAATATAAGAACTGAATTAAAATGGTCCCAATTTATGGCTCCTCCTCTGTAG

**Locus Pma21**, motif AAAAC (reverse complement: GTTTT)

>IFVYH3A01BLUTF length=636 xy=0541_3505 region=1 run=R_2013_09_11_14_57_29_

GGCAATCTCGTGTCTTCAGTTTGCCTGACTGTGGGCCTTCAGACTGTGGGAGGAAAACAAAGCACCCAACTGGCAGACCCCCACAGGTGTTTTGTTTTGTTTTGTTTTGTTTTGTTTTGTTTTGTTTTGTTTTGTTTTGTTTTGTTTTGTTTTGTTTTGTTTTGTTTTGTTTTGTTTTGTTTTGTTTTGTTTTGTTTTGTACTCGGGCACCACAGTTACTGCAGGTGCATGCATAAATGTACATGAAACTGATTATGCTATAATTCTTGATCTCTGTAGCTTTATAGATTTTAAGATGAAATAATTCATATAAATCAGTAAACATGTCAGATGCATCCAGAATGATGAACACTGCAGATTATAAAACTGATCCAGAATATACAACATTGAGAATGGCTCCTATTTCATATCACAGTGGTCATCAACTGTCTAGTCAGATGCTATTGTAAACAGATAATATAATTTACCGTATATTATATACCCTGTTGGTGTTTTCAGTTGAGAATGTGCTATTCGTCCCATTCTTGCTTCAGCTGCTCAGCAGCTCATACACTGCGCTTCACAATGCACCAGATGTTTTGAGTGGGACGACAGCTCTGGACTGCAGACAGTTCTGTCCAGCACCTGCACTCTTGT

**Locus Pma22**, motif TCTCC (reverse complement: GGAGA)

>IFVYH3A01DAE6M length=520 xy=1231_3020 region=1 run=R_2013_09_11_14_57_29_

GTAACGGTTGTGGTTTGAATGAAAGTCTTAGGATCTGTATCCGTTATGGTTGAATATGAATATAACAGAACCCGGATGTTGTTTGAAAGGAAGCGTATTTAAGCGCCGTTATTCTTTTCCATTGTAGGCTATATAAATGCCTTTGCAGGAGCCGCGCGCGATGCCGCGCAGCAGAGGCATCCGCGCGAGGCTGAACTGGAGCTGAGGGCGCTGTGCAGCTCAGCGAGGCGCTCGCGCTGCCGGTTTAAGACTGCTGCTCGCGCGGCACCGAGTACAGCGGAACGAGGCTTCACGGAGAGAAACGACTGAGGCGGATGGGGAAGATTAAAGGAATACAAGAATAAGAATGGAGAGAGGAGAGGAGAGGAGAGGAGAGGAGAGGAGAGGAGAGGAGAGGAGAGGAGAGGAGAGAGTGATGGAGCTACGAGTCTTACCTTCAGCAGCTCGCGGACTCGGCGAGAGCGCAGAGCGAACAGCAACCGGATAAGCGCTAGCTCTCTCACTCACTCTCTCACTCTCT

**Locus Pma23**, motif AATAG (reverse complement: CTATT)

>IFVYH3A01BTI3F length=284 xy=0629_1113 region=1 run=R_2013_09_11_14_57_29_

GTCCTTGGCAGAACTGGGTGTGTGATTTTATCACACACAAACACAATCATAACTGCTTTTCCAATATGGGCGGAAAGACAAGGATATCCAAACGGAGTGAAAAAGTCATCCTTTCACCATTTCCAAATTATTCTATTCTATTCTATTCTATTCTATTCTATTCTATTCTATTCTATTCTATTCTGATAATCGTATTTTAGCTCAAAGCTAAATGCATAGTCTGTCTTTGCCCATAGCTAAAACATATCTTAGCTCAAAGTTAGGCTTAATCTTAATGCAAAGCT

**Locus Pma24**, motif ATATT

>IFVYH3A01A27RR length=699 xy=0329_2181 region=1 run=R_2013_09_11_14_57_29_

GTTACCACTGATGCAGCCCATATATGAAAACATGCAAAAACAGCTTGTTTTGAAATCACCAATTATATTATATTATATTATATTATATTATATTATATTATATTATATTATATTATATTCTCTGTTGTCTTTTATTTGGGACACTGTTCTAATCTATACTGGGTAAGACCCCACTTTCTTCCTAGAACAATCTGAACTCTTCAGAGCATGTATCTCTCAAGGTTCTTAGATATTCTGCTCCATGTAGGGATGACTGCGTCAGGTAATTGCTGCGGATTTGCCAGCTGTGCATTCATGCCATAAATCTATTTGATTCAGATCTGGTTGACTGAGGCAACTAAAGTACACTGAACTCATAGTCAGGTTCATGAAACCAGCTTGACTTGTGGATCATCATGCTGGAAATAACTATTGGAAGAATAAACGATAACCATTAAAGGGGATGCACATATGCAGCAACAACTCTCAGGCAAGCTGTAGCATTCAAACAATTCTCAACTGTTATTTAAAGACCCAATGAGTGCCAAGAAAAACATTTCCCACACCAATACATCACCAATCCCAGCCTGAACTGTTCGGCCAAAGTTGAGTCCAAATTTGGACTCTAAGATCACAGCATTTCACAATAGAAATCCAGATTCACCAGACCAACCAAGGTACAAATTGCAGTGGGGTTCTGAGAGGTTTCATTACTTATAA

**Locus Pma25**, motif AAGGC (reverse complement: GCCTT)

>IFVYH3A01B67BV length=736 xy=0785_0073 region=1 run=R_2013_09_11_14_57_29_

CTATAGTGTTGCAGTCATGTTCTGGTAGTGGACTAGGTTTCTCAACCTGGTGGTTCTGGCTCGGATGTTCCGGTACCTCTTTCTGGATGGCCGGAGGTTGAAATGGGAGGGCGGGGTGTGTGGGATCTGTAGGTATGTTGATGCTTTCCTCAGACGGCTCTTGACGTAGATGTCCTGTATGGGTGGGAGGACAGCTGCGGTGATGTTACCACACCCCGCAGGGCCTTGCATGATGCAGAGACGGAGAGCGAGGGGTCATGATTCAGATAAGCGCTTTAAGCTGAAAAAGATTCTTTTCAATTGATCCTTTCTCCACAGCGACTTTGGATCACTTTCAATCTCTTCAGCATGTGGCCTTTCGGCGATGCAAATGCTCCGTGACATTAAAATAGCCTGCTGAAGGCAGAGAGAGCGGCGCTTAGCCACAGAGTGAGAGCATGCCCAACAGCACTTCAGTGCATTCTTTAAAGGGGAAAGAAATCCAGGCGGAACCACTTTCAGGTTCTTCTGTAGGGCAGCAGTTCACAGACTGCACACAGGGCTCGCTTTTCACTGTGTCCTTGCCTTGCCTTGCCTTGCCTTGCCTTGCCTTGCCTTGCCTTGCCTTGCGGATTATCTGTTTCTGATGCCAAGCAGAGCTTTTAGGCCGTCGGTATGAACCCAGGATTCCTACATCCTCATCTCCTAAAAATGGCAGAAGCACATCCAGAATGTACCGGAACTCTTATCTTCGCTG

**Locus Pma26**, motif ATCTC

>IFVYH3A01CQFO1 length=875 xy=1004_0355 region=1 run=R_2013_09_11_14_57_29_

GCTTGCTCAAAATGCATCAGCCTGTCACCCCAATAGGACAAAACACTGGGTGTGTAATACACCAGTGCTGATCTCTCTTTGCTGGCTTCCTGTGACCTACAGAATCAACTTTTAAAGACTAGTGATGACTTACCAGGCTTTATACAACAAGGTCCCAGCATACAACACTGAGCTCGTATGTTCATAATCATAATGATGTTTCCTCCCCTCACCCTGAACCAAATTAAAGTCAAAGAGGGGCAGGCCTTTGCAGGTTACACCCTCTGACATCAGACATTCAGCTTTGGCTCATCTCATCTCATCTCATCTCATCTCATCTCATCTCATTTTATCTCATCTCATTTTATCTCATCTCATCCTTCTGGGTTGTGAGAGGTGCTGGAGCCTACCCCAGCGTTCAATGGGCAGAGAGCAGGAAACACCCTGGACAGACAGGCTTTGGCCTAAGTCCATAAAAAACTTTTAAAAATCTTTTAAAGAGATTCTTAATATCTTGCTTTTAGTAGTTCCTGAACATTTATTGAACTGTTTATTGGTTAATTTTGCATTTCATTGTTTTAGTGTAAAGTGCTTTGAGCCTTGTTCTAGAAAAAGTGTTGTATATATATTTATCTGATTATTTAATATTCCATAAAATTTATACCTCCTGACTAAATTCACCTAGTTGAAGTAACTTCAAAGCTTATTAAACGATAAAAAAACACTGACTTTTCTTTAAAATGTGCATTGTATGTAAGTGAAGCACTTAAGCAGTAGACGCAGATACCGGCTTCCCCCGTCAGTACAATTCAATGCATATTCATGTAGAATTTCTGAAATTTATATTCATGAGGAATGCAATTTCAATATGACCTTAGGGAGCATACTGAAGAGGG

**Locus Pma27**, motif TCGGC (reverse complement: GCCGA)

>IFVYH3A01BEX9V length=621 xy=0463_0881 region=1 run=R_2013_09_11_14_57_29_

AAAGAGAGAGAGAGAGAAAGAGAGAGAGAGAGAACCGTACTCCTCCTAGAGTGAAATGTCCAGCACCCACTGACCGGTCACTATCCACTGACCACGATGAACTGTTCAGTGTTATAAACTACACACAACTGTAACACCAGGTACTGAATAATTCATAGTAGGTACTGTATGATTCATAGTGGATCTAAATACTACATCATATTAAATTAGTTAAAGGATTTTTTACACACAGTATATGGATATTATAATATTAGCCCCGCCCCTCCGGGATTGTGGCTGTGGCTGTGTTACTGCTGGGCCCGTATCTGACCTGTTCCATCAGCAGAGGTTCAGAGAGGGCCATGACGGGCTTAAGGTACAGCACCACTTCCATTCAGCCCACAGAGGAGCTGGGCCGGGCCGAGCCAAGCTGGGCCGAGCCGAGCCGAGCCGAGCCGAGCCGAGCCGAGCCGAGTTGAGCCGGTCCGGTCCAGTCCGAGCCGAGCCGAGCCGAGCCGAGCGTATGGGCAGTGAATGAATAGCAAGTACATTAAGAACAAAGAGTGCTGACCCTTCTGTCTGCCCTCAATCGCTCGCTCTCTCCCTCTCTCCTATTCTCTCTCTCCTATTCTCTCTCTCCTA

**Locus Pma28**, motif AAGTC (reverse complement: GACTT)

>IFVYH3A01B7P5E length=670 xy=0790_3984 region=1 run=R_2013_09_11_14_57_29_

GTTAAATATCTATGCACCTTACACTCATACCTCTACTCAGAACTGAGTCTTATTTATTGTACTGTCTTACACGTTGCATTTTCTTAGTCTGTCCTGTACTGTAATGTTTTTTATTCTCGTTTTGTTCCACGTGGCACCCTTGTTCTGAAAAAAATTTCATTTCATTTGACTGTGTACTTGTACAGAACTTAATGACAATAAAAGCCACTTGACTTGACTTGACTTGACTTGACTTGACTTGACTTGACGTCATGTTACACATTACCTATAGATTTGTGAAGAATAACAAAAGTCCACTGTCTCTTTTCTTTTTCAGCTCAGGATTTCATCTGTGTTTCATTCACATGTATGATTTTGCTTATTTTTAGAGAGCTGTGAAAATGGAATATAAATGGATTACATTTCATATTTTAGTGAATATATCATGATTGACAACATGTGGTTTGATGCTCTATAGCAGCCATTTTGTAGATGCATTGTTCATGAAAAACATCACTGGTTCAAATAAAAACAACCCATCTTAATCATAACCTAGTAACATAAGGTTATGCTGACCTAAATTCAGTTTAGACAATGTTCTGTGTTGGAGAGGTTTCGCTGTTTTCTGATGGAGTCATGTGATAACCCGGAGGAGTAGGATAACACCTGTGAGGAAAAGTACAGTAAAACA

**Locus Pma29**, motif AGGCT

>IFVYH3A01EM54J length=670 xy=1787_0049 region=1 run=R_2013_09_11_14_57_29_

GATGTTATTAAAGTATATTAATATTACTGTAACACTCATAAACACATGTGCAATAGCATAAGGATCATAAATCAGTGCAGAGGCTAGGCTAGGCTAGGCTAGGCTAGGCTAGGCTAGTTGGTATTGTGTGGAGAAGCCTATTTTGGAAAGTGCTCGTAGGCTGAAGGTTTGAACAGGCTTAGAACTAATATTCAAATTAGTGATTTGCGTAGAGGAATACAAATGCACGATTTGAATGTAGTTGGCAAACAGAAGCGATACAGAGGTGCACATGCTTTCACAGGAGGAAGCCGTGTCATAAGTCATATGCAAGAGTTGGAGCACGCCCATTCGTATGACGGGTGAGGTAGATTTTGAAAATGAAAGGGAAAATAAGTAGGGCTGCCGTCTAGAAGTTGAATCCTCAGTCGGAGACCCTTTAAGAAGACTGAGACCCTTCAAGGCGACTGACAACAGTGACTCCTAAAACAGACTAAAGCTTACAAAGTTGTACAAGAACTCCACAAATGTAAAAATACACTTTAATAATTATTAAAAGGGGAATAATAAATATATATTAAAGTTCTAGCTGGGCTGTGATGCATGCTTGCTGGCGGTTGCTTGACGTGCGCTGGCGGTTCACCGCTGAAGGAAGCTGAAGCCGTGAAGCTGAGACACACGGGGGGAGCGA

**Locus Pma30**, motif TCTCC (reverse complement: GGAGA)

>IFVYH3A01EBX15 length=777 xy=1659_0667 region=1 run=R_2013_09_11_14_57_29_

AACGGTAACGGGGAGAAGGAGGAGAAGAACACCAAACAGGAGCAGAGGGTGAGAGGAGGAGCAGCTGAAAGAGGAGGGGAGAGGAGAGGAGGACACAGGAGAGGAGAGGAGAGGAGAGGAGAGGAGAGGACACCTTACTTTGAAAAGCCCTTTCAGCATGATGTCGATGATCTTGTACTGCTGGTTGACGTCGTTGAGCTCCACCAGGTTCTTCAGAGCGTTCAGCTGATCCTTCCTCTTGGTCTCGAACAGACGCTTATCTGAAGCTCAGCTCAGGAAGAAGATCCACCACCCAATTCATATCTACTCAGCTTACGGAGTCACAGGGCTGCAGATGTGACTGATTGGCATTAGGAACATTCACACATCTGGACTGGCCTACCAAATCCCCAGATGTGAATCCTCCTGATAATCTGTGGGACCTCCAGGGCAGGCATCTACCACACAGGAGTGTCTGAACACTGAGGCCTGCATCCCTGAACGTGACTGATGCAATGCCATGTGACGCGATGTGATGAGATGAGATGTGACGTGACGTGACGCGATGTTGTGTGATGTGATGCGGTGCGGTGTGGTGGGTGCGGTGCGGTGCCAGCAGCCTACTAGACAGATTAACAGTACAGGACAAATCAGTGCCAACTACACACCGTAGACACACGTACACCTGAGGATACAGATTTCCAGGTTGGAGTCGTGCCTGTGCCGGTGCGTGTGAGGGTCCGAGTCTGTGGAACTCAGCAGCAGTGTTTGCTGCCAGCAGCAGCACGGTGCACACAC

**Locus Pma32**, motif ATCT

>IFVYH3A01CUVBU length=549 xy=1054_2440 region=1 run=R_2013_09_11_14_57_29_

TCTAAGCCGAGTGTACTCTGGCAGTAAAGGAGGGCCTGCTCATGCAGACAGCTGCTGACGCTGTGGCAGTCAAATACAAAAGGAGCTAAAAACATTCCGCCTAGGCCAAATTCCTGCCACGAATACACTGTGTGATTTTGGCTCCTCTGTAAACACAAAGTCACGGTCATAAGCTCTTTCCCAATGGGAACATTCAGAGGTGGAAAAGACACAACAGCGCCCCCATATAGGTGGAAAACGGTGAAGAAGTCGAAGTCCATATATACTGACTGTACTCTGTGCAGTCTCTGTCATTCATGAATTTCCCTCTGGGATCAATAAAGTATCTATCTATCTATCTATCTATCTATCTATCTATCTATCTATCTATCTATCTATCTATCTATCTATCTATCTATCTATCTATCTATCTATCTATCTATCTCTATTCATGCAGTACAAGTTAAAGGGTTCACTCTGACTGTGGTGTTGTGTTGACACGGCATGGCTATTAGCGACCTCCCCCACTGTTCTCCTGTACTGACTCACGTGCCGCTCTTCTCCGCTGCT

**Locus Pma34**, motif ATCT (reverse complement: AGAT)

>IFVYH3A01D0B3G length=875 xy=1526_3754 region=1 run=R_2013_09_11_14_57_29_

CTTAATACCAAGCTAGCTTCTTCTTATAGCTACCTAAAAACTTATACTAGAAGCTAGCTTGGTATTCCCAGCATGGACATTATAGCGAAGGATGCTAAGTGCTGCTAAAGCTACAGCTACAGCTAACAGCTTCAGAATCAATGCAATGTAATATGGAGAATGGCGTCACATGCTCATATGAGTTCATATAAGATATCATGTTCACACTCCTTCCGAGTTACATGGCGCTATAGCATGTACTACAGGCCCAGAGTGCCGATGACAGAGACGCCATTCTCTATATTACATTACAATGATTCTGAAGCTATTAGCTGAAGCTTTAGCAGCACTGATGTACACAACCAGAATCATATCCATGATAACAGTCTATTCCATAAAATAGATATAGATAGATAGATAGATAGATAGATAGATAGATAGATAGATAGATAGATAGATAGATAGATAGATAGATAGATAGATAGATAGATAGATAAAGGGCACTACAGGGCATCTTGGTTCTTGTTAAGTTTAAGAGCTACATGAGAAAAACATCTTCATACTGTAAATTCATTTTCATGCTGAGATTGTCTGTGCAATTTTGAACAGCATTGAAAGAGGACTAACCTCCTCAGACCTCTGTCTGGCTGGTTAAGCCCACCTGTGTATTTAGATTCAGCATACAGACCTTTCTAATTCCCAGCCCCACCTACTCACCAGCCCTGTATTTAACCCTGCCCTGTATTGCCACAGGGGACCTAATGGAGGAGCTGGCTTTCAGAACACAGAGCCAATGAGCAATTCAGGCAGACTGGAGGGTGGAGGCCCCTTTTTCCATTATGGCCATAAGGAATAATCATATGCAATAAGCACATGAAATAATAATGTACTGATCG

**Locus Pma35**, motif ATCT

>IFVYH3A01CYVFW length=525 xy=1100_0794 region=1 run=R_2013_09_11_14_57_29_

CAAAAGCAAAACTGCAGACTTCGTTTTGCTAGCTGTCTATTAGGACTGAAAGATTGCAGAATAGCAGAATCGCAAAAAAGCAAGAGCTACAATTTTTCTAATAGTCTCTTATCATCTACTAAAAACAAATATCAAGCAGGACAGAGTAGGTAGATGAATTAGAACCCATGTGCTCTGTTCATCAATAAGTAACTGGCAGTCTGGCATTTTAGTGGCTACATTAAAGCCCCAAGCCAGAAAAAGTGGCTACAGTAGACTTCTATATAATAGATCTATCTATCTATCTATCTATCTATCTATCTATCTATCTATCTATCTATCTATCTATCTATCTATCTATCTATCTATCTATCTAATGGACAGTTCAATTGGTTCTAATGAACCTGCCCTTTGCCAAAGCTAATGGTCTTTGCTAATGCCATTTAGTTTGACGTATGAGGTCAGTGTTTAATTGCTATGAAGTAACTCCAGTGATGCGAGATGTGGTTGGTTTTTCCTCAGATATGAATGAGCACAGTTCCAGGT

**Locus Pma36**, motif ATCT (reverse complement: AGAT)

>IFVYH3A01CKBIV length=451 xy=0934_1733 region=1 run=R_2013_09_11_14_57_29_

CTGGGGATCATGAGACAGAAGGTTACAGTGGTGCAGATGAAATGACTGAACGCTATGCAGAACAGGGGGCAGAGGGACATAGACGGCCAACTTCTTTTTAGAAAAAGAAGTGTAGGTAGTGGGGGTGCTGTTTCTAATTATATCCTCCTATACTGCTATTTTTATTTTCTCCTCTGACAGTGTAATATTACAAACTAATATTAATAATCATACAAAGAAGAAGATGAATTTGACCCATTTTCATGATTTTTTGTTTAAATGAATATCATGATGAAATGCCACACCATTATATATGGGTATGTGTCCCACTATGATAGATAGATAGATAGATAGATAGATAGATAGATAGATAGATAGATAGATAGATAGATAGATAGATAGATAGATAGATAGATAGGTGCCTAAGTTCACGTGCAAAACAAAAGGGCTACACTGTATTGATCAGGCTGGG

**Locus Pma37**, motif ATCT (reverse complement: AGAT)

>IFVYH3A01B2405 length=617 xy=0738_2983 region=1 run=R_2013_09_11_14_57_29_

AGTCTTTCTTGGGTTAGTCACTTACGATGAACGCTGAGAGAGCTGACAGTGCGGTTATGAATGAGTTACTATAAGACTTTTACCATGGAGTGACTGGGTCGGCTGGGTCACTGTGGTCTTCACAGTTGTAGTGGGGGTCTTGGTTGTAGTCCCTGTAATATCACACAAATACAGCATCACAGATAGATAGATAGATAGATAGATAGATAGATAGATAGATAGATAGATAGATAGATAGATAGATAGATAGATAGATAGATACTCTATTGATCCCAGAAGGAAATTCAGAATAACACAGCACCATACGCTTTGCACATATATGCTTGTTTGTTTAGTCCATTCCTTGCAAGGCATTGCTGAATTTCCATTTGCATACTTAGCGTTTTCCTTGTTGACTGTTTTAGCCGTGTTTGACCTGTGTCTGGTTTTTGACCTCTCTTTGATTGGACTGTTTGCACCTTTGGTTTTGACCCTAGCCTGGTTTCCCACCTTTGCCTTCTGTCTGCCCCATGTTTCTGGTACTGGTTCTAATGGTTCTGAGCTGGATTGGCTCTTCTGGACTGTCACTTTGTAAATAAACCCCCTAAAATATATATAGATATATAGATCTATATATA

**Locus Pma39**, motif ATCT (reverse complement: AGAT)

>IFVYH3A01EAUNO length=728 xy=1646_2850 region=1 run=R_2013_09_11_14_57_29_

GGACTGCTATCCCGCTCTGAGAGATTCTGGGTTGATGTATGTAATAATTAGTCAAAATGGTATTATTTTGAACAAAAACCGTCATAGAGTTTAGTTAATTAATAAATAAATAACTTAATTAATGAATTAAGTCAGCTAATACGTACGTTGTCTTTAAGGATCAAGGATAATTATTTTCAAAAATTAACAAATAAATAAATAATGTGAGAAAAAAAAACCAATGACCTGTTTTCTACATTTGGATAGATAGATAGATAGATAGATAGATAGATAGATAGATAGATAGATAGATAGATAGATAGATAGATAGATAGATAAATGTAAATAAAAACTATTTTTTAGACTCCAGTTAAACTTAAACCCGTCGTAATTCTAGTCGTAAGGAACAATCGCAGGTCCTTAAAGACTTGCGCGCTGACGTCACCACGCCCGTACGTGCTATGTCATCGCCACTGCACTTCCGCCATCCGGGTAGTAGATTTTGAGGTAAGTACAGCTGGACTTCTGTAGCTTTGTTTTTTCGCGGTTTTAGAGATGAATTCGATTATCTAGCTTATTAATTCACCGTGTCCATGTTAATTGTCCGTGTCTGTGTCAGGACTGTTAGATATGTCCGTGTCTTTGTACCGTGTCGCTCTNGTTAGAGCCTCGCCTGTGTGTTCGTTCATTATCAGGATGCTATGCTATGCTATGCTAGCTGTGCTATGGCTAATCTACTCAGCACGAGC

**Locus Pma40**, motif ATCT

>IFVYH3A01CWZ1K length=583 xy=1078_3558 region=1 run=R_2013_09_11_14_57_29_

AGGGTGATGTGAGGCTAAATGTCCCCTAAGGAAACTTATTTTTCACATCATATTGTTATTAATACGACATATATAACTTTATATAACAAAAAAACCTCGGAAAACTCAAAAAAAGAATAGTCGTGCATCTTCACTGGTTGTCGTGGAAAATAATTACTTTGGGTAGATTTGTGTTGCAGACCTTGTAAACCACTGGTTACCCACCACTGTCGCTAGCTTGCTCTAAAATGGCCCATTTCACATCAAACAACTATGAATCTATCTATCTATCTATCTATCTATCTATCTATCTATCTATCTATCTATCTATCTATCTATCTATCTATCTATCTATTAGGTTATATTGTAACTCGTACTACTTACTCTAGGTTTGTCATATGTATGAGGTGTGCCAGGTTGATATGTTCTATGCACGTGATGAGATCACACACCTGAACACAATTCAAACCACTAGAGCGAGGAGGAGTACAGAACGTCTCCAAATGGCAATGTGTGGGCTCGCTGGGTGCGTCTGCAAGCGTGTGTGTCTGTGTGCGTGTCTGTTGTGTGTGTGTGCGTGCGTGTGTGTGTGTGTGTGTGTCTG

**Locus Pma41**, motif ATCT (reverse complement: AGAT)

>IFVYH3A01B6LX5 length=283 xy=0778_1035 region=1 run=R_2013_09_11_14_57_29_

GATATTATTGTAACTGATTATATTGTTTCAAAATCAAAATAGTTGAGTCTTCACTTTCAGACTGATGCATCACAATACATCACAGGTAAATAGATAGAGAGATAGATAGATAGATAGATAGATAGATAGATAGATAGATAGATAGATAGATAGATAGATAGATAGATAGATAGATAGAGTTGNAAAATCATGGAAATGTTGGAAAGCGTATAGCTGAGCAGCTCAATGGAAGGGAGGCCGTTGTGGTTCTCTTAGTATTAATAGTTAATGACATAATTATCTT

**Locus Pma42**, motif ATCT (reverse complement: AGAT)

>IFVYH3A01E2NHQ length=484 xy=1963_1500 region=1 run=R_2013_09_11_14_57_29_

GTTTCCCTTCTCCCAACTTTTTTAAGATGCTCAGTGAGTTCATAATTTTCATGAAACGGTAAAATGACTTTCAACATCAGATATGTATTCCATGTTGTACTGTAAATAAAATATGGGTCTATGAAATTTGAAAATCATTGCATTCTGTTTTTATTTACATTTTACACAGCGTCCCAATTCCTATTTTTTGGAATTGGGGTTGTTGATAGATAGATAGATAGATAGATAGATAGATAGATAGATAGATAGATAGATAGATAGATAGATAGATAGATATCCAAACAGGCTTGGCATTAAATACACCTTGTGAATGTCAGTCTGTCCATCTGGTGGAGATGAGGACTGGTAGGACAATCCCTGCAGCAACATTGAAATCTGTGACGATGGTTAACGCTAAGATAAAGGCATCAAAAGACAGATTGATATTGAAACCGAGGGCTACTGCTAGCGGGATGGCTTGGAATAAGAATATCAGGAGACTAAA

**Locus Pma43**, motif ATCT (reverse complement: AGAT)

>IFVYH3A01E2MSB length=372 xy=1963_0585 region=1 run=R_2013_09_11_14_57_29_

TACAGATCAACACAGCTGTATGAATGATTAAGTCACTACTGATTTGGAACCTGAGATGTGGTAATGTGCATCACATGTATGTAAGTTATATTGATAAAAATATTTCTTATTTGTATTGTATGTGTAAGAGCCAATTTGTAAAGACAGAACTGTACAAAACTAAACAGATAGATAGATAGATAGATAGATAGATAGATAGATAGATAGATAGATAGATAGATAGATAGATAGATAGATACTTTATTGATCCCAGAGAGAAATTCAAGAAAGATACACACAAACAGGCCTGGCAACAGCAAGAGACATAATGACTGACCTTTCAGAGGGTTTCCAGATATGTAAACAGAGAGAGAGAGAGGAGAAGAGAGAGAG

**Locus Pma44**, motif ATCT

>IFVYH3A01BZSN7 length=741 xy=0700_2645 region=1 run=R_2013_09_11_14_57_29_

ATTGAATTGAAAAACTGGAATAAAAAATCAAGTTATAATGAACTAATAAAGTAATAATCTTTTTTTTTTACCTTCAAACCATCAAACATGAAGCTATATTTGGCTGTAGTTGCTAATGTAAAGGGTAGACTCTTGTAAGACAAAAACCGCCTGTTTCTTTGTTCATTTACTTTCAAAACCTATTTGTTGGACTTTTGTCGTTTGTGTATCTGTACTGAAGTATTCCTTTTTACTCTAATTCCACTTTCCATTGTGTGATTCTGTCTTTCTTTTGCCTTATGTGCGCATAAATTTGCTAAATTTCCTTCGGGATCTATCTATCTATCTATCTATCTATCTATCTATCTATCTATCTATCTATCTATCTATCTATCTATCTATCTAAAAACGCACCACATCAGAACACAAGAAGCACAAAGTGAGTCCAACCAATCAAGGCACATTGTTTTGAGCTTGTTTTGACCTGTTGGAAATAATGGAAACAATTATTTTGTGCGTATTATCATTTTTAATAGACATCAGCGTCAGACTTTTAACTGATTGGACTAAGAGAGACACTAGAGTCTTTTCACCTACAATAGGTTGATTAAGGGTCTTATTTTAGAAATGACAATAGAGCCATAATATATCAAGATACTTTTACTTTGCTATAACCAAGTACATCTGAAGGTACATACTTTTGGTCCAAAAGTCTCTGAATGCAAAATATTTCATCACTGAAATGTCCATGCTCTTCTCTAT

**Locus Pma45**, motif ATCT

>IFVYH3A01CS5JC length=756 xy=1035_0182 region=1 run=R_2013_09_11_14_57_29_

CTGCATGGGATGGATTATCACAGGACTCCATTAGGAACCTCTACAGCTCCATGCTGCGTTAGCAAACATGGCTGCCCGATGCCAAACTGAGAGACACTACTTCTGTATGTGTAGCTCAATAAATATTACCCATATCAGTCTAAATGTATCATCATGTATTGTTCCTGATATACTTTATCTTCCTTCCCAACAGCACTGCACTCCCACACCTCCTTCTGGATGCAGCTGTTCTTTTGATGATGAGTGTATATTAAATACATACAAGATCATTTTACCTGCCTAGCTCTCGTTGTTAGCAGTTTCCAGAGCATTCATCTTTGACAGAGAGAGGATGTTTCAGATATGCCTGACTGAGTTTGCTGTTTCTCCATGGTTTATTCACGAGTGACTCCATCACTGAGCTGTTGGGGCAGCTGTGGATCAGGTGGTAGAGCAGGTTCTCCACTAACCGCAGGGCTTATGGTTCAGTCCCCAGGTCCTCTTGTTCATGGCTGAAGCATCCTTAAGCAAAGCATCCAAATGCTCTCTGAGGACATTCAGGACATAGTCAGGACATTCATACATCAAAAATGCTTGTGGTGAGGCTCAGGTTATTACTCTACAGTATCTATCTATCTATCTATCTATCTATCTATCTATCTATCTATCTATCTATCTATCTATCTATCTATCTGTCTATCTATCGAGTGCCGTAGTAGTGGCGAAAGGCTCACAGTTTGTTCAGGAACCTGATGGCCTGTGGGTAAAATCTAGTCT

**Locus Pma46**, motif ATCT (reverse complement: AGAT)

>IFVYH3A01CHXX4 length=673 xy=0907_1418 region=1 run=R_2013_09_11_14_57_29_

GCTGAACATTAGATGTGTTTGTTCACACATGCTGTTTAAGGAGCAAAAGAGCGATTAGAAGCAGAATTAGACACACACTGAACTCTCTGAGCGTCCGCACAGTGAGACCTGTTACCTTCAGCACCACACACACACACGCACTGAGTATTTGTTTCTGTGAATGGTGGGGACATTACATAGACTTTCATTCATTTTCTGGAGACCCAAACCATAACCATAACTACTATTAGCTTAACCCTTACTACCTTAACCTCAACCTTAAGCTTACCCTAACCATAACCTTAACCTCAACCTTATCTTAACCCTACCCCTACCTCAACCTCACCTAACTCAGAGGTGGAACATAGATGTTTGTTTATTTGAGCCATTGATGTAAACATCTCATTGCCGAGAAACAAATGTTGTAATGGTCAAATATTTATGATAGATAGATAGATAGATAGATAGATAGATAGATAGATAGATAGATAGATAGATAGATAGATAGCTTTAAGTTAGTGTATTTATGAATATTGAGCGACTTTACGGACAGAACCTCCAGCAAAACCTTAGCAGACAGTGCATTCCTAACATCCTAACTTGAACAGAAAACGAAAAATTACAAAATAGCCTTTTATTGGTTTGGGNTGCTTGTAACGGATCCATCAACAGACACCTGAAGTGATATGAGAGG

**Locus Pma47**, motif ATCT

>IFVYH3A01B83HN length=454 xy=0806_2393 region=1 run=R_2013_09_11_14_57_29_

ACTGTGTAGCTGTGTAATTTCTACTGGCTGCTAAATTAAATCCTTTGGAATCAATGAAAGAATCTATCTATCTATCTATCTATCTATCTATCTATCTATCTATCTATCTATCTATCTATCTATCTGAATCTAAATGGTGCCAGACCAAATTTGATATAAACATAAATCTCTAGTGCACCATGTGACAAAAATACAGGGGCCTCTGCTGTGGAACGGTTTTGCTTGTTCATGCACTTCAACAACTACACAAACACACATCGTCGCTGACTAAAAAGAAAAACTAGGATCTACCATACAGAAACTTTACAGGAGAGGTCATAAACCTCACTGAACTTTAATGCAAGTCAATGGAACCAGATTTTTTCCAAGCAGTTTTGGGCCGTTTCTGTTGGTCCATCCACCATGACATTTTTCACAGAATGTTAACAGCAGGTCATAGGATCAAATGATGCAT

**Locus Pma49**, motif ATCT (reverse complement: AGAT)

>IFVYH3A01CMMZ6 length=662 xy=0960_3428 region=1 run=R_2013_09_11_14_57_29_

GGCTGGTGTGTTAAGAAGTAGTCGGCCCTCACCGCACAGTCTTACAGTATCAGCCTGGGCACTCCCCTATGGCTTTCTGACTGGGAGTCACTATTAGCAACAACACACTTCACGTGTGTCTGGCATCTGGCCCATAGCTTTAATATAATGCTTCCCACATTAGCCTCATGGTCACACACACACAGAGCTTCAGCCTGGCTCTGTCTCAGTGGACAAGTGCAGCCCAAGGCTCTTCGTGCATGTAGGAACAGATGTAGCCATTGCAGCACATTAACACATTTGACGTCCATGCACTAATACATTCATCCCCGGCTGACCATACGTTCAGTGGTCACTTCCATCTCATAGATAATCTCTTACCTTATAGTGGGGACTGCAAATACAGGGCAGGTGTTTCTAATAAAGTGGCAACTAAGTGGAAGCATATGTAGGTTGTTTATATTACAGTGGCTAGTGATTGGAAATACAAGGCAGGTGTTTCTAATAAAATGGCCACTGAGTGGAAATGTCTATGGATATTCATATTAAAGTGGCCAGTTGCTGCTTATATGGATAGATAGATAGATAGATAGATAGATAGATAGATAGATAGATAGATAGATAGATACTTTATTGATTCCAGAGGGAAATTCAAGAATATGAGGCAGCTGTTTCTAATAAAG

**Locus Pma50**, motif ATCT

>IFVYH3A01AVDLS length=499 xy=0240_1038 region=1 run=R_2013_09_11_14_57_29_

GTGTGCGTGACCAGTGATGATTCTGACTCGGGGAAGAACGTTGAGCTGCAGACCAGAGAGATGATCGAGTGGGCCAGTGCAGGATTCCTTCCTACCGGAGCCAAGGGCCTGGAGCCCCCACCAGGTAACACATTCAACACACTGTCATACTCAGCATCTCCATATACACTGTGTGTGAAATACCTGCCTGGCTATCTATCTATCTATCTATCTATCTATCTATCTATCTATCTATCTATCTATCTATGTATCTATATCAACCCTTGATATTTATTTGGATTGAGGGTGGTGCTCATTTTCCAACTGAAAGATTAAACTGGAATTTGATTCATGTTTAGTAGCATAAGGGTGTGTCTCATAGTGTTGTGTGTGTGTGTGTGTGTGTGTGTGTGTGTGTGTGTTGTGTGTGTGCGTGTGTGTGTGTGTGTGTTGTGTTGTGTGTGTGTGTGTGTGTGTGTGTGGTGTGTGTGTGTGTGTGTGTGTGTGTGTGTGTGTGTGT

**Locus Pma52**, motif AGCC (reverse complement: GGCT)

>IFVYH3A01CUSWG length=566 xy=1053_3390 region=1 run=R_2013_09_11_14_57_29_

TTGCTGCATGCTGCATGTAGCCAATCGGGCAGGAGAAGGTTGAGGCGAAGAGGTAAGTGGACACGTACCATGAGACATTAGGTACCTGTATGTTTGTAGACATTTCATGGAATGACTGAAACTCTTCAGATGACTGCTTCCCATCACCACCTCTGTGGACATAAATTTATTTATTTATTATTTAGTTCATTTTATAAGATAATCATTTTGCCGAGTCAGTACTTCCTTCTGTTCATACTGTACAACCCCGTTATTGTATTACTGTGGTGCATTGTGTAACAGCTACTGGCTGTTAAATTTCCTTGGGATCAATAAACTGACCGACGGACTTGCTGGCTTGCTGGCTGGCTGGCTGGCTGGCTGGCTGGCTGGCTGGCTGGCTGGCTGGCTGACTAGGCAGTAACAAAGTGCAGCAGACTGCAACTTTACTGCAATGAACGCTGCAGTGGCCAATTGGGCGAGAGTATGCTGGTACGTCCTACAAGCATCACACTAAAAACAAACCCCTTTCAAACATGGAGGAATCGCTAATGTAAGCTAGCTAGCTAGACAGCTAGCTGGTATAG

**Locus Pma55**, motif TTC

>IFVYH3A01C2SS1 length=433 xy=1144_3779 region=1 run=R_2013_09_11_14_57_29_

GATCTTCATTCTGCAACTAATTTATTCATTTAACAATTTCCCTCCAGAAGTTGGAGCAGAAGGCTCTGAAGCAACCTAAGCAGAAGAAATCTAAGTCGGCTGAATTTCTGATGGGTGAGCTCCGTATGATGAGTTATTGCTCATAATTTGTGTAAGGATTAATTAACTAATTACAGACAAATGGCTCTGGGCTAAATTTTGCTCTTGCTTGTCAGGGCGCAATTTGTAATTATTTTAATCATTTCTTCTTCTTCTTCTTCTTCTTCTTCTTCTTCTTCTTCTTCTTCTTCTTCTTCTTCTGTTCCTCCTTATTCATTTTGTCTTTACCTTTTTCTTCTTCTCCGTTACCTCTCTTTTTGTGGTTGGGTTTTCAATGTTATCCTAATCGTGCTACAGTGACATTTCTTGGAGGCTTCGCTTGAGTGTTTCAACT

**Locus Pma56**, motif ATT (reverse complement: AAT)

>IFVYH3A01DVV25 length=673 xy=1476_1183 region=1 run=R_2013_09_11_14_57_29_

GGAAGGGAGCTTAAGAAAGGTGGTTGCTAAAGTGGTGTATTGTAGAGTGTCCAGAAACATTCTGTGTCTTATTGAGCATCCAAAAAATGTTTTGGATACAGACTGTATTGTGACTGTGATGGAATAATACATACAGGTAAAATGCATGAGGTATATGGCATATATAGAATTCATGGGGTTTCATATGGTTGAACAGTCCCCCATTCATTTCCTATGGGGAGAAAAAAAAAATCGCTTCAATGAATATGGTGTGAGAATCGTCCAGAAATCCAAAAAGCACATATGCACTACACCTGTATATGATGAACATTTGGGCCAGATTTGGTGCCTGTAGCTGGGAAATTGTGGGCTGTGAAATAGGAACAGAATTTGAGCCTAGAAAAATAATAAGAAGAAGATTATGAAAACTTTAAGCCAACTTAAATAATAATAATAATAATAATAATAATAATAATAATAATAATAATAATAATAATACTTAGGAGATGGAATTAGTGCACCGACCGTGTTCCGCATTGCCATAGCCCAAGATTTTTGGTGGAGGCCCTTCCAAATCCAATTCTTAATTTGTAGATATTTGTTTGAAACAAAGTTAGTCGTAATGCCATTATACTCCATCACGCCGTTCCCGCATGTTCAGATCAAACAATTACGGAGTGCCCGATGTTGCTGT

**Locus Pma57**, motif AAC

>IFVYH3A01B3JQR length=402 xy=0743_1569 region=1 run=R_2013_09_11_14_57_29_

CCAAGTTCACCCATGTCACTCCACTGCTATGCTCCCTTCACTGGTTTCCTGTTGCTGCCTGCATTAGATTTAAAATTCTGATGCTCGCCTACAAAGCCAAGAATGTACCAGCCCCTCCCTACCTTATGGCAATGGTTAAGGGTCGATCTGTACCTTGAGCCCTTTGAGCTTCGCGTACAGCTCGACTCAACCGGCCATCCTTGAAAAAATAACAATGAGGATCCTGATCATTGTGACAACAACAACAACAACAACAACAACAACAACAACAACAACAACAACAACAATAATAATAAATCTTCTGTTATGGAAAAAAAATATTTGTAAATCTGCACAAACAGGGGCTTTCAGAGTGGTTAAAAGCACTTGCATTCCATGAACCTCAAACACTATTTTTCCCCC

**Locus Pma59**, motif ATATT (reverse complement: AATAT)

>IFVYH3A01C5UJM length=527 xy=1179_2640 region=1 run=R_2013_09_11_14_57_29_

AAGGAATGTTCTCTATTTACTGTGGTATTTGTTTAGACTTTGGAGCAGAATTAAACATTCTCATAATTATACTCCTTGTCCAGCTCTGAGAGTCACTCCTAAGGATTTTCTGACAGCTACATTTGTAAAGTCTTTCAACATGAATCACTTTGCAGTATGCTAAAGAGCTGCTGCATTTCTGCTGCCAGTTAAGTCACATTAACTCTTACAAAAAAAAATTGACAAAGACCAGTAGCCATGATTTTTAAATACCACTAAAATATTATTAAATGGTCTTCTTAAAAATATTTATCAACTTTGATAATATAATATAATATAATATAATATAATATAATATAATATAATATAATATAATATAATATAATACTGTATAATGCTGCCAAAATGACAAAACTATTACATCCAAAAAATGTGTTAAACTGCCAAGTCTTCTGTACAGTGCACAACAGTGTTAGCTTGCATGTAGCAATTATGAAAATTAGCTTTTATTTAACATGTGGTAAACTCTTTTAACTAAGACTTTCATG

**Locus Pma60**, motif TCTCC (reverse complement: GGAGA)

>IFVYH3A01DAE6M length=520 xy=1231_3020 region=1 run=R_2013_09_11_14_57_29_

GTAACGGTTGTGGTTTGAATGAAAGTCTTAGGATCTGTATCCGTTATGGTTGAATATGAATATAACAGAACCCGGATGTTGTTTGAAAGGAAGCGTATTTAAGCGCCGTTATTCTTTTCCATTGTAGGCTATATAAATGCCTTTGCAGGAGCCGCGCGCGATGCCGCGCAGCAGAGGCATCCGCGCGAGGCTGAACTGGAGCTGAGGGCGCTGTGCAGCTCAGCGAGGCGCTCGCGCTGCCGGTTTAAGACTGCTGCTCGCGCGGCACCGAGTACAGCGGAACGAGGCTTCACGGAGAGAAACGACTGAGGCGGATGGGGAAGATTAAAGGAATACAAGAATAAGAATGGAGAGAGGAGAGGAGAGGAGAGGAGAGGAGAGGAGAGGAGAGGAGAGGAGAGGAGAGGAGAGAGTGATGGAGCTACGAGTCTTACCTTCAGCAGCTCGCGGACTCGGCGAGAGCGCAGAGCGAACAGCAACCGGATAAGCGCTAGCTCTCTCACTCACTCTCTCACTCTCT
